# Ethnobiology of snappers (Lutjanidae): target species and suggestions for management

**DOI:** 10.1186/1746-4269-7-11

**Published:** 2011-03-16

**Authors:** Alpina Begossi, Svetlana V Salivonchyk, Luciana G Araujo, Tainá B Andreoli, Mariana Clauzet, Claudia M Martinelli, Allan GL Ferreira, Luiz EC Oliveira, Renato AM Silvano

**Affiliations:** 1 Fisheries and Food Institute (FIFO), UNISANTA, Santos, SP, Brazil; 2 UNICAMP (Capesca and CMU, CP 6023, Campinas, SP), Brazil; 3 CPDA-Rio de Janeiro (CNPq, PD), Brazil; 4 ECOMAR, UNISANTA, Santos, SP,11045-040, Brazil; 5 Institute for Nature Management, National Academy of Sciences of Belarus, 10 Fr. Skaryna Street, Minsk, 220114, Minsk, Belarus; 6 Natural Resources Institute (University of Manitoba)Canada; 7 Depto. Ecologia/UFRGS, CP 15007, 91501-970 Porto Alegre - RS, Brazil

## Abstract

In this study, we sought to investigate the biology (diet and reproduction) and ethnobiology (fishers knowledge and fishing spots used to catch snappers) of five species of snappers (Lutjanidae), including *Lutjanus analis, Lutjanus synagris, Lutjanus vivanus, Ocyurus chrysurus*, and *Romboplites saliens *at five sites along the northeast (Riacho Doce, Maceió in Alagoas State, and Porto do Sauípe, Entre Rios at Bahia State) and the southeast (SE) Brazilian coast (Paraty and Rio de Janeiro cities at Rio de Janeiro State, and Bertioga, at São Paulo State.).

We collected 288 snappers and interviewed 86 fishermen. The stomach contents of each fish were examined and macroscopic gonad analysis was performed. Snappers are very important for the fisheries of NE Brazil, and our results indicated that some populations, such as mutton snapper (*L. analis*) and lane snapper (*L. synagris*), are being caught when they are too young, at early juvenile stages.

Local knowledge has been shown to be a powerful tool for determining appropriate policies regarding management of target species, and artisanal fishermen can be included in management processes. Other suggestions for managing the fisheries are discussed, including proposals that could provide motivation for artisanal fishermen to participate in programs to conserve resources, such as co-management approaches that utilize local knowledge, the establishment of fishing seasons, and compensation of fishermen, through 'payment for environmental services'. These suggestions may enhance the participation of local artisanal fishermen in moving to a more realistic and less top-down management approach of the fish population.

## Background

Reef fishes of the Lutjanidae family (snappers) are important targets for fisheries in several regions worldwide, including Australia [1], the South Pacific [2], Africa [3,4], North and South America [5,6], and Brazil [7]. Snappers are locally called "Vermelhos" or "Pargos" in Brazil and are commonly exploited by artisanal fishermen [7,8]. There are about twenty-three genera of snappers (Lutjanidae) and the genus *Lutjanus *includes more than 70 species [9]; in http://www.fishbase.org[10] there are 173 scientific names listed for *Lutjanus*. In Brazil, there are twelve species of snappers from five genera: *Etelis oculatus, Lutjanus analis, L. apodus, L. bucanella, L. cyanopterus, L. griseus, L. jocu, L. purpureus, L. synagris, L. vivanus, Ocyurus chrysurus, Pristipomoides freemani, P. aquilonaris *and *Rhomboplites aurorubens *[11,12].

Snappers have been intensively captured by marine fisheries on the northeastern Brazilian coast [7,13,14], but these fishes have also been caught on the northeastern Brazilian coast by artisanal fisheries using mainly hooks and line and/or gillnets [15]. Snappers are carnivores, and species live in reef environments along the NE Brazilian coast at different depth ranges [16-20]. For example, at Porto Seguro, on the NE Brazilian coast, 38% of 352 fish landings have caught snappers [8], a target also of the artisanal fisheries on the northeastern Brazilian coast [7]. Nevertheless, some species of snappers may have been overfished in Brazil. For example, *Lutjanus purpureus *has shown a decrease in the catch per unit of effort (CPUE); there was additionally a decrease in the weight and length of captured fish, indicating an increase in the capture of juvenile fish [21]. An analysis of the fishing time series of 1967-2000 indicated the vulnerability and local market extinction of snappers in two states (Rio Grande do Norte and Pernambuco) on the northeastern Brazilian coast [19]. Another study [22] indicated that the yellow snapper, *Ocyurus chrysurus*, and the vermilion snapper, *Romboplites aurorubens*, which are two commercially important species for the Brazilian coast, have been overexploited. Most of the Brazilian fish production comes from artisanal fisheries [23]. Therefore is important to address the importance of artisanal fishing in tropical countries, especially in Brazil. Data from 2002 [15] showed that the contribution of artisanal fisheries to the total catch is 88% in NE Brazil, 34% in SE Brazil, and that the contribution of artisanal fisheries has increased in SE Brazil since 1980.

The importance of managing fisheries resources has been emphasized, considering the current threat to marine resources [24-26]. Observing and measuring marine resources is costly [27], and there is an urgent need to obtain data on marine tropical fisheries [28]. Data are especially lacking for rocky and reef fishes that have slow growth and late reproductive maturity, including groupers and snappers [29]. This study was motivated by an urgent need to improve our understanding of the biology of snappers, and data were gathered based on the knowledge of the scientific community and that of local fishermen. These data may be useful for improving the local management of snappers. When paired with scientific knowledge (published literature), local knowledge could improve our understanding of high-biodiversity systems where basic biological information is lacking [30,31].

Studies that have combined scientific knowledge and the knowledge of local fishermen have been useful for enhancing the dialogue between resource users and managers. In some regions, such as tropical developing countries, these studies may be the only available source of knowledge about exploited fishing resources [28,32-34]. There is evidence that even artisanal fishing can impact fish populations, especially populations with late maturation and slow growth [35]. Reef fishes, including snappers, are among the fish species that are more vulnerable to fishing pressure [29]. The study of snappers in Brazil could be improved by including methods of ethnobiology, which is a discipline devoted to the survey of local ecological knowledge held by local people, including fishers [36,37].

The importance of using local fishermen's knowledge as a tool for fishery management has been acknowledged, analyzed and applied by a variety of researchers in many parts of the world, including the Pacific and small-scale Asian fisheries [38-45]. One study [46] applied both scientific and local knowledge to research and to the management of lobster fishing off the coast of Maine, USA, supporting an example of integrative management (co-management) where fishers are active participants in the lobster management. Another study [47] analyzed the definition of local knowledge and its implications for the management of several different extractive and agricultural communities in many parts of the world, including fisheries. In Brazil, local ecological knowledge related to small-scale fisheries has been studied by several authors [31,33,48-53]. Nevertheless, in Brazil, local, ecological knowledge of fishermen has not been fully applied to fisheries management, mainly because of misunderstandings on the part of environmental government agencies and biologists about of the importance of this information. Therefore, information gathered from fishermen can turn them active participants in management processes and it can be useful in places where there is lack of scientific data, such as many tropical fisheries.

Our results addresses the dialogue between scientific and local ecological knowledge [32,33] by studying how snappers are being caught in the Brazilian coast, and by getting information on its diet and reproduction. An increased vulnerability of snappers on the coast of Brazil is observed, coupled with an urgent need for knowledge about their biology. The methods used here could be applied elsewhere, given the widespread exploitation of this vulnerable group of reef fishes.

The main objectives of our study were a) to record and analyze data on the snappers' reproductive period and diet through direct biological observations; b) to record and to analyze the same kind of data gathered by interviewing local fishermen; and c) to compare both sources of data (scientific and local knowledge) and suggest potential applications for improving snapper research and management; d) to suggest management of snappers through both scientific and local knowledge, using social-economical-ecological tools, such as co-management through fishing agreements and payments for environmental services.

### Study sites

**Figure 1:**
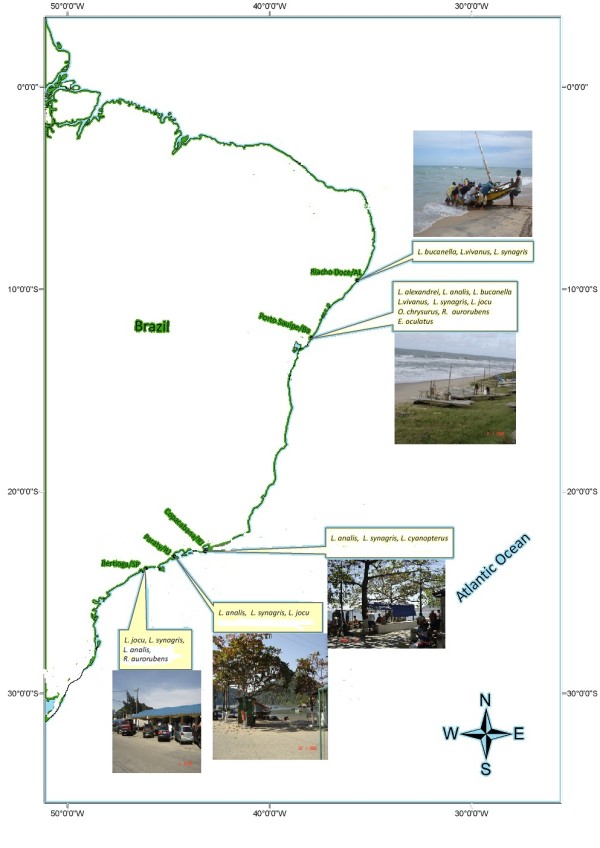
**Study sites in the coast of Brazil and snapper species**: a) Porto Sauípe, bahia; b) Riacho Doce, Alagoas; c) Praia Grande, Paraty, Rio de Janeiro; d) Colonia de Pescadores de Copacabana, Rio de Janeiro, and Bertioga, São Paulo.

The five sites that were studied were located in northeastern ('Região Nordeste') and southeastern Brazil ('Região Sudeste-Sul') of the Brazilian Economic Exclusive Zone (EEZ) [54], as follows (Figure 1): Riacho Doce, Maceió, Alagoas State, and Porto Sauípe, Bahia State in northeastern Brazil; Paraty, and Copacabana (Rio de Janeiro city), Rio de Janeiro State, and Bertioga, São Paulo State in southeastern Brazil (Figure 1). The continental shelf is narrower in northeastern Brazil compared to the southern Brazilian coast, which implies that there are differences in artisanal fisheries. For example, on the northeast coast, fishermen work near the end of the continental shelf, locally called (in Porto Sauípe, Bahia) "*paredão*" (big wall). These fishermen can catch fish that are usually found in deeper waters, such as snappers, which are usually caught with hooks and lines on rafts (*'*jangadas'). The fisheries studied were artisanal fisheries that use small boats or rafts and catch snappers mostly with hooks and lines, but some fisheries often use set gillnets.

### Northeastern Brazil

#### 1. Riacho Doce

Maceió, Alagoas State: This is a small community that is located close to other small fishing communities, such as Garça Torta, in the municipality of Maceió, the capital of Alagoas State. Riacho Doce is a tourist site where local fishermen divide their time among fishing tourism related activities and other jobs. Eleven fishers' rafts ('jangadas') and two fish stores, where fish are caught and sold, were observed in Riacho Doce.

#### 2. Porto do Sauípe, Entre Rios

Bahia State: The village of Porto Sauípe is a small fishing community located in the municipality of Entre Rios, about 80 km from Salvador, the capital of the Bahia State. This village has about twenty-five fishermen listed in the Colonia de Pescadores Z-28 (a local fishermen's association), and about fourteen rafts ('jangadas') that are used for artisanal fishing were found in the villages.

### Southeastern Brazil

#### 3. Paraty, Rio de Janeiro State

The municipality of Paraty includes approximately thirteen small-scale artisanal fishing communities from the northernmost part of Tarituba to the southernmost part of Trindade [55]. The community includes about eleven local fishermen. Fishermen from nearby Araújo Island land usually fish at Praia Grande [55].

#### 4. Copacabana Beach, Rio de Janeiro, Rio de Janeiro State

Copacabana beach has one of the oldest artisanal fishing communities in Rio, which is associated at the Colonia Z-13 [56]. Based on earlier research projects [36,48,49], we estimated that about twenty-five fishermen frequently land their catches at Copacabana beach.

#### 5. Bertioga, São Paulo State

Bertioga, which can be easily reached through the Rio-Santos highway, is a small city that includes about twenty-five fishermen. Bertioga has been a part of earlier projects describing the ethnobiology of artisanal fisheries [36,53,57,58].

All of the Brazilian artisanal fisheries described above commonly sell and catch many fish species, including bluefish (*Pomatomus saltatrix*, Pomatomidae), cutlassfish (*Trichiurus lepturus*, Trichiuridae), mackerels (*Scomberomorus *spp., Scombridae), mullets (*Mugil *spp., Mugilidae), groupers (*Epinephelus *spp. and *Mycteroperca *spp., Serranidae), snooks (*Centropomus *spp., Centropomidae), weakfishes (*Cynoscion *spp., Sciaenidae), as well as rays, sharks (many species of Chondrichthyes) and many other species. We previously observed that snappers are very common in the fishery of Porto Sauípe, Bahia compared to the other sites that were studied.

## Methods

At Riacho Doce the local fishermen estimated that about twenty artisanal fishermen live in Riacho Doce. The snappers were sampled in the 'Peixaria do Haroldo' (fish market). At Porto do Sauípe we interviewed twenty-two artisanal fishermen in this village during a project conducted in 2005 and this data set was used as a baseline for the current study. Ethnobiological data about coastal fishes are available for this village and adjacent fishing communities [36,53]. The snappers were sampled in the 'Peixaria do Chico' (a small fish market). Local fishers used hooks and line to catch snappers at depths of about 165-220 m (75-100 'braças', a local measurement adopted by fishers). In Paraty, we studied the snappers and the fishermen that land their catches in the fishing community of Praia Grande, close to Araújo Island. Our study of fishermen and snappers was performed especially at the 'Peixaria do Sinésio' (a small fish market), located at Praia Grande, among other fish stores from Paraty. At Rio de Janeiro, our study of fishermen and snappers was carried out at the local landing point, where fishermen and middlemen sell their catch directly to consumers. At Bertioga, our research was conducted at the main fish market and landing point. We collected snappers mainly at two small stores in this fish market (Figure 1).

All snapper species were obtained from fish landed by fishermen, mostly between April and November of 2008. *L. analis *was collected mainly from April to July in Copacabana, Rio de Janeiro, with a similar pattern observed for *L. synagris *in Bertioga, SP.

Complementary methods were used to collect data on snapper biology and ethnobiology:

### 1) Sampling of snapper stomachs and gonads

During each trip to the studied fishing communities (described above), we searched for the landing/market point where fishermen landed snappers. All of the available snappers that were found in these landing points/markets were sampled by either buying the fish (which was then opened up for analysis) or its contents (viscera). Each sampled fish was weighed (g) and measured for total length (TL) (mm). The gonads (volume) were measured in milliliters (ml) and visual inspections were conducted to document the gonads' color and the presence or absence of visible eggs (macroscopic analyses). These procedures followed methods detailed in earlier studies [33], which have been used for other coastal fish species[36,37].

Based on gonad volume, regardless of the presence of visible eggs or sperm, the measurements from 288 fish were collected and used to calculate the gonadossomatic index (GSI) for 241 snappers. This index was calculated based on a classic formula [58] and used in studies on artisanal fishers [37] as: (GSI = [gonads weight/body weight] × 100). The weight of the fish gonad was defined by its volume, assuming the average density of fish flesh was 1.065. The volume data were standardized, and gonads with less than 1 ml of volume were considered to be equal to 0.5 ml. Seasonal differences in the fish GSI were investigated with a non-parametric Kruskall-Wallis test.

### 2) Field trips

the number of field trips to collect data was different for each study site, but monthly trips were performed to Bertioga and Copacabana, SE Brazil. For the NE Brazil areas, we made a total of three field trips; one to Riacho Doce and two to Porto do Sauípe. Although the second trip to Porto do Sauípe was not planned in our project, this trip was made necessary based on information provided by the fishermen about the spawning period of snappers. The goal of the trip was to double check the gonad maturation season of the studied snapper species (the second trip occurred in October 2008, Table 1). The number of days and of collection of snappers fieldtrips varied as a function of the distance of the field sites from our main institutions (first author). For far places, such as Alagoas and Bahia, we had to concentrate data collection in one or two trips. For nearby places, such as Rio and Bertioga, we could perform monthly visits. Paraty was included later, as a way to compare data between Copacabana (Rio) and Bertioga, an in-between site.

**Table 1:** Snappers (Lutjanidae) sampled in 2008-2009 in the Brazilian coast.

	Season/Month												
				
Site	Species	Autumn		Winter			Spring			Summer		Total	
		AP	MY	JU	JL	AU	SE	OC	NO	DE	JA		
**Bertioga**	*L. analis*		*2*	1								**3**	
	*L. jocu*	*1*	*2*		3							**6**	**44**
	***L. synagris***		*4*	15		3	5			2		**29**	
	*R. aurorubens*			6								**6**	
**Maceio**^**1**^	*L. buccanella*										5	**5**	
	***L. synagris***										15	**15**	**28**
	*L. vivanus*										8	**8**	
**Paraty**	*L. analis*					5			4			**9**	
	*L. jocu*								1			**1**	**44**
	***L. synagris***					3			31			**34**	
**Porto Sauípe**	*E. oculatus*				2							**2**	
	*L. analis*							1				**1**	
	*L. alexandrei*				7							**7**	
	*L. buccanella*				5			2				**7**	
	*L. jocu*				1							**1**	**137**
	*L. synagris*				7			1				**8**	
	***L. vivanus***				14			15				**29**	
	***O. chrysurus***				57			9				**66**	
	***R. aurorubens***				4			12				**16**	
**Copacabana**	***L. analis***	*8*	*9*	3	5		1	4		2		**32**	
	*L. cyanopterus*		*1*									**1**	**35**
	*L. synagris*			2								**2**	

**Sub-total Total**		**9**	**18**	**27**	**105**	**11**	**6**	**44**	**36**	**4**	**28**		
		**27**		**143**			**86**			**32**		**288**	

^1^Riacho Doce, Maceió: one *L. analis *was observed, but not collected.

### 3) Interviews

interviews with fishermen were based on standardized questionnaires with a few questions about snappers, such as their occurrence at the study site, their diet and their period of reproduction. The interviewed fishermen were selected based on previous interviews from earlier projects in Bertioga, Porto Sauípe and Copacabana beach [36,37]. In the other study sites (Riacho Doce and Paraty), fishermen were opportunistic selected at the landing points. Interviews were done with full-time, skilled fishermen who had lived at the sites for at least ten years.

### 4) Identification of the fish and stomach contents

The collected snappers were identified in the field using identification keys [10,11,59], as well as Plates I-V from Western Atlantic, Fishing areas 31 and 41 (ftp://ftp.fao.org/docrep/fao/009/ac481e/AC481E49.pdf). Visible food items found in the stomach of the snappers were collected for identification at Capesca (Unicamp) using taxonomic keys [11,12,60]. Identifications of fish found in the snappers' stomachs contents of snappers were made by experts in the field from MZUSP (fish: R. Caires and J. L. Figueiredo; crabs and shrimp: G.S. Melo).

### 5) Weight-length relationships

The relationships between weight and length were calculated for all collected snapper species, in g and mm, respectively. These relationships were described by second-order polynomials. A linear approximation of the Weight-Total Length (W-TL) relationships did not seem informative because this procedure assumed *de facto *that the mass of a fish was linearly proportional to its length. While this assumption may hold true for some length intervals, it fails for the whole range of lengths. In this study, therefore, the approximation made with the second degree polynomial provided a much better fit than the linear one (we explored the possibilities of a better fit by comparing the determinacy coefficients R^2^, for different fits).

## Results

### Snapper species caught by fishermen

A total of 288 snappers were collected and 86 fishermen were interviewed over 142 days of fieldwork from April 2008-January 2009 in the five studied fishing communities (Table 1). These collected snappers belonged to four genera and ten species of Lutjanidae (seven species are from the genera *Lutjanus*) (Table 2). About 90% of the total number of snappers collected belonged to just five species of Lutjanidae: *Lutjanus analis *(45 individuals), *Lutjanus synagris *(88), *Lutjanus vivanus *(37), *Ocyurus chrysurus *(66) and *Rhomboplites aurorubens *(22). Among those fishes collected in Bertioga and at Riacho Doce, Maceió, the species *Lutjanus synagris *(66 and 54% of individuals caught, respectively) was predominant. In Copacabana, *Lutjanus analis *(91%) was predominant, while in Porto Sauípe the most frequently caught species were *Ocyurus chrysuru*s (48%), *Lutjanus vivanus *(21%) and *Rhomboplites aurorubens *(12%) (Table 2). A greater diversity of snapper species was found in the fish landings of the fishing community at Porto do Sauípe, Bahia (Table 2, Figure 1).

**Table 2:** The most caught snappers in the study sites in Brazil

Period	*L. analis*, Copacabana	*L. synagris*, Bertioga	*L. synagris*, Maceió	*L. vivanus*, P. Sauipe	*O. chrysurus*, P. Sauipe	*R. aurorubens *P. Sauipe	*L. synagris*, Paraty	Total
**April**	8							**8**
**May**	9	4						**13**
**June**	3	15						**18**
**July**	5			14	57	4		**80**
**August**		3					3	**6**
**September**	1	5						**6**
**October**	4			15	9	12		**40**
**November**							31	**31**
**December**	2	2						**4**
**January**			15					**15**

**Autumn**	17	4						**21**
**Winter**	8	18		14	57	4	3	**104**
**Spring**	5	5		15	9	12	31	**77**
**Summer**	2	2	15					**19**

The seasonal occurrence of the five snapper species (*L. analis, L. synagris, L. vivanus, O. chrysurus and R. aurorubens*), based on collections during the whole year, was as follows: 21 individuals were collected in autumn (April-May), 104 in winter (June-August), 77 in spring (September-November), and 32 in the summer (December-January). In Bertioga and Copacabana, snappers were collected during the whole year, while in Porto Sauípe, they were collected only in the winter and spring. In Maceió, the collection was made only in the summer and in Paraty, the collection was made only in the winter and spring (Table 1).

**Figure 2:**
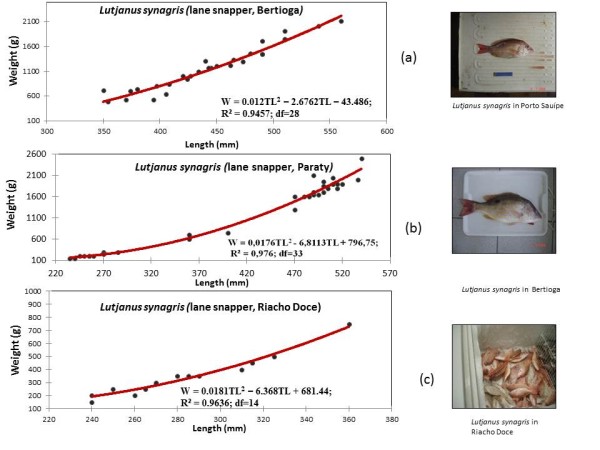
Weight and length of *Lutjanus synagris*, caught by artisanal fishing, in Bertioga (São Paulo), Paraty (Rio de Janeiro), and Riacho Doce (Alagoas).

**Figure 3:**
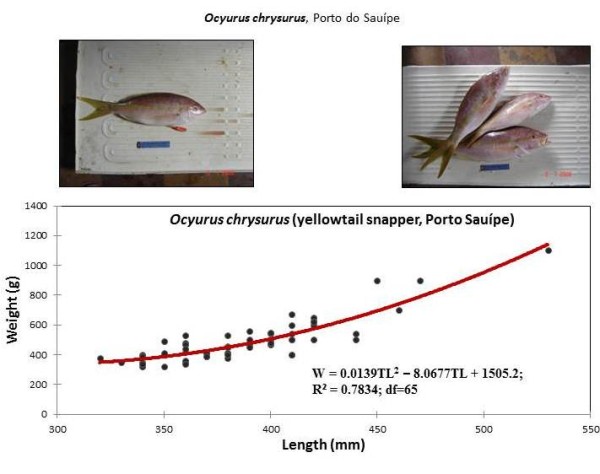
**Weight and length of *Ocyurus chrysurus*, caught by artisanal fishing, in Porto Sauípe (Bahia)**.

**Figure 4:**
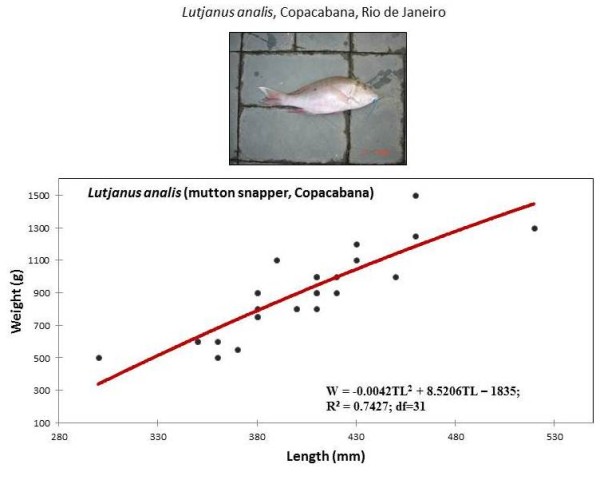
**Weight and length of *Lutjanus analis*, caught by artisanal fishing, in Copacabana (Rio de Janeiro)**.

**Figure 5:**
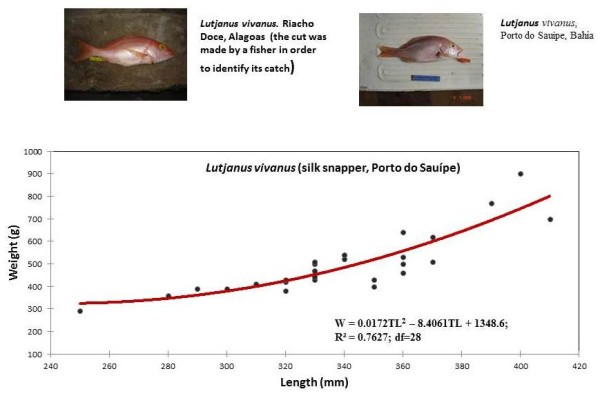
**Weight and length of *Lutjanus vivanus*, caught by artisanal fishing, in Porto Sauípe (Bahia)**.

**Figure 6:**
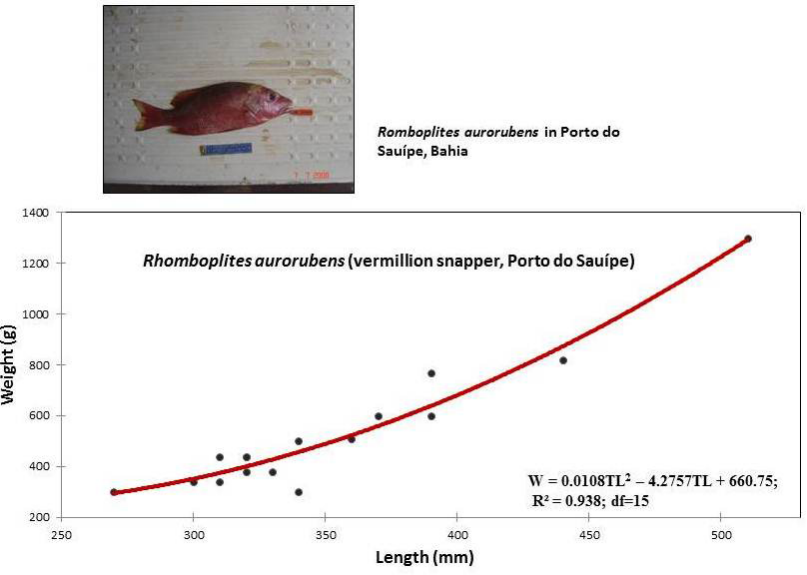
**Weight and length of *Romboplites aurorubens*, caught by artisanal fishing, in Porto Sauípe (Bahia)**.

The five most collected snapper species were analyzed in detail below. They are listed in order from the most individuals to the least individuals collected (Table 2 and Figures 2,3,4,5, and 6):

### *Lutjanus synagris *(Linnaeus, 1758)

A total of 88 lane snappers (*L. synagris*), locally called 'vermelho-ariocó', were sampled in all of the five studied fishing communities. However, 89% of the sampled fish were collected in Bertioga (SP), Paraty (RJ), (SE Brazil) and Riacho Doce (NE Brazil) (Table 2). Lane snapper was associated with reefs, and this species formed large reproductive aggregations and fed on small fishes, crabs, shrimps, worms, and gastropods, among other things [10]. The identification of lane snappers was based on the presence of ten spines and twelve rays on the dorsal fin, along with a silvery-reddish body color, longitudinal yellow stripes and a diffuse black spot above the lateral line [11] (Figure 2). Additional information obtained recently in a current project confirmed the relative importance of lane snapper, amongst the other snapper species, in landings of artisanal fisheries in Praia Grande (Paraty).

### *Ocyurus chrysurus *(Bloch, 1791)

66 yellowtail snappers (*O. chrysurus*) were collected in Porto do Sauípe, NE Brazil, mainly in July 2008 (86%) (Table 2). Yellowtail snapper is locally called 'vermelho-guaíba' or just 'guaíuba'. This fish is a reef species, which lives in coastal waters and formed aggregations. The yellowtail snapper feeds on fish, crustaceans, worms, gastropods and cephalopods [10]. The dorsal fin has ten spines and twelve to thirteen rays as well as a body with a yellow band that goes to the caudal fin [11] (Figure 3).

### *Lutjanus analis *(Cuvier, 1828)

Most of the 45 mutton snappers (*L. analis*), which were locally called "vermelho-cióba" or "cióba", were collected in Copacabana beach, Rio de Janeiro (71%) in 2008. At other sites, this fish was collected mostly in the autumn and winter seasons, especially at the sites of Bertioga, Paraty, and Porto do Sauípe (Table 2). Mutton snapper, which is now considered to be a vulnerable species by the UICN red list, lives in the continental shelf close to islands, forms small aggregations, and feeds on fish, shrimps, crabs, cephalopods, and gastropods [10]. Its body has a dorsal fin with ten spines and fourteen rays. It has a lateral black spot below the first rays of the dorsal fin as well as pale-blue stripes below the eyes [11] (Figure 4).

### *Lutjanus vivanus *(Cuvier, 1828)

The silk snapper, *L. vivanus*, was collected in NE Brazil, mainly at Porto do Sauípe in Bahia State (78% of 37 fish). This fish is locally called true snapper" ('vermelho-verdadeiro', or 'vermelho-legítimo', or 'vermelho-comum', or 'vermelho-original') in Porto do Sauípe. This fishing site has a relatively narrow continental shelf, allowing fishermen to use hook and line at large depths, which probably helps them catch silk snappers, named as a "reference fish" (prototype) within the local nomenclature of snappers. This fish is abundant around the Antilles and the Bahamas [10]. The species is common on shelves, but it can be found in water deeper than 200 m. Silk snappers feed on fish, shrimps, crabs, and other invertebrates. It reaches about 500 mm in size, has ten dorsal spines and fourteen rays in its dorsal fin, and a reddish body color [11]. Local fishermen in Porto do Sauípe, Bahia consider the yellow pigment in its iris to be a typical feature of this fish species (Figure 5).

### *Romboplites aurorubens *(Cuvier, 1829)

The vermilion snapper (*R. aurorubens*), which is locally called 'vermelho-prumirim' or 'paramirim', is found, on rocks, gravel or sand [10]. This snapper species forms large schools and feeds on fishes, shrimps, crabs, and other invertebrates. It has twelve spines and ten to eleven rays on the dorsal fin. The body of the vermillion snapper is reddish with dark oblique stripes on its dorsal part and yellowish stripes can be seen below the lateral line (Figure 6).

The other snapper species that were collected included *Etelis oculatus *(Porto do Sauípe, Bahia), *Lutjanus alexandrei*. This fish was first identified in the field as *L. apodus*, but revised to *L. alexandrei *after *pers. comm*. by J. L. Figueiredo, and consultation to reference [59]. The fish was collected in Porto do Sauípe, Bahia, *Lutjanus cyanopterus *(Copacabana, Rio de Janeiro), and *Lutjanus jocu *(Bertioga, Paraty and, Porto do Sauípe) (Table 2).

#### Weight-length relationships of snapper

Weight-length relationships were calculated for all collected snapper species and are described by second-order polynomials in Figures 2, 3, 4, 5 and 6. Among the collected snapper species, the greatest deviation from linearity was found for *Lutjanus analis *(Figure 2), and the smallest deviation was found for *Lutjanus vivanus *and *Rhomboplites aurorubens *(Figures 5 and 6). The precision of the approximations used for all of the presented experimental data was high, so one can assume that there is a strict functional relationship between fish weight and length. This trend was more evident in *Lutjanus synagris *and *Lutjanus analis *(Figures 2 and 4). The largest difference in weights that corresponded to the same fish length was observed for *Ocyurus chrysurus *(Figure 3).

The regression coefficients and values of the weight-length relationships for *L. synagris *suggested that body depth (or vertical length decreases as fish grow (Figure 2), but this trend was not as steep as the trends for other species like *Lutjanus griseus *(Starck and Schroeder, 1971). We observed that the sizes of this fish species caught by fishermen from Bertioga, Paraty and Riacho Doce, Maceió ranged from 250-550 mm (maximum length is 600 mm TL [10]).

**Figure 7:**
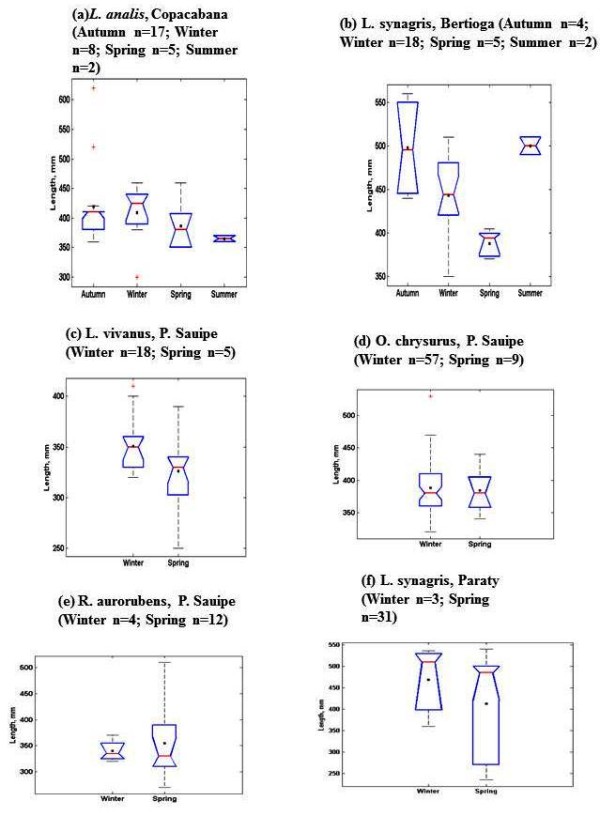
**Length distributions for species of the Lutjanidae family in different seasons of the year**. Samples were taken in April 2008-January 2009. black dot - mean value; top and bottom of lines - maximum and minimum values; above and under the line of blue figure - 75th and 25th percentiles; red line - median; red cross - stray values.

**Figure 8:**
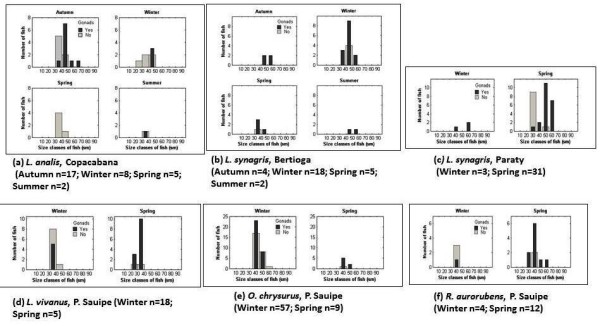
**Seasonal variation in distribution over size classes with or without visible gonads of snappers caught by artisanal fisheries**.

Samples of *O. chrysurus *included mostly fish landed by fishermen at the Porto do Sauípe. These fish had body lengths between 350-450 mm (the maximum length recorded was 863 mm, [10]) (Figure 3). The mutton snappers, *L. analis*, which were caught by fishers in Copacabana, were between 350-450 mm (TL) (Figure 4). The silk snappers, *L. vivanus*, had a range of body lengths between 300-380 mm TL (Figure 5). The *R. aurorubens *that were caught were measured between 300-350 mm in length (Figure 6). The seasonal length distributions of these snapper species are shown in Figures 7 and 8. We observed the highest snapper patterns in length for the autumn and winter seasons.

#### Reproduction: gonad analysis of snappers obtained from fish landings

The analysis of fish gonads was possible only for five of the collected species, which were *L. analis, L. synagris, L. vivanus, O. chrysurus*, and *R. aurorubens *(Table 3). Visible eggs in the gonads of females were observed in autumn for *L. analis*, year-round for *L. synagris*, and in spring for *L. vivanus*, *O. chrysurus *and a few *R. aurorubens *individuals (Table 3). It appeared that most of the studied snappers reached sexual maturity during the spring (September-December) (Figure 7). The GSI values of the five most frequently caught snapper species showed some seasonal differences. For example, *L. analis *collected in Copacabana showed a higher GSI in summer than in spring. Additionally, *L. synagris *collected in Bertioga showed a higher GSI in summer than in winter (Table 3). Two snapper species collected in Porto Sauipe, *L. vivanus *and *O. chrysurus*, had higher GSI values in the spring than in the winter.

**Table 3:** Percent of snappers with visible eggs in different periods of the year^1^

Period	*L. analis *Copacabana	*L. synagris *Bertioga	*L. synagris *Maceio	*L. vivanus *P. Sauipe	*O. chrysurus *P. Sauipe	*R. aurorubens *P. Sauipe	*L. synagris*, Paraty	Total
**April**	12.50*							**12.50**
	*0*							*0*
**May**	0.00	50.00						**15.38**
	*11.1*	*0*						*7,7*
**June**	0.00	13.33						**11.11**
	*0*	*0*						*0*
**July**	0.00			28.57	1.75	0.00		**6.25**
	*0*			*0*	*0*	*0*		*0*
**August**		0.00					0.00	**0.00**
		*0*					*0*	*0*
**September**	0.00	20.00						**16.67**
	*0*	*0*						*0*
**October**	0.00			73.33	66.67	8.33		**45.00**
	*0*			*6.7*	*22.2*	*41.7*		*20.0*
**November**							38,70	**38,70**
							38.7**	*38.7*
**December**	0.00	100.00						**50.00**
	*0*	*0*						*0*
**January**			73.33					**73.33**
			*26.7*					*26.7*

**Autumn**	5.88	50.00						**14.29**
	*5.88*	*0*						*4,8*
**Winter**	0.00	11.11		28.57	1.75	0.00	0.00	**6.73**
	0	0		0	0	0	0	*0*
**Spring**	0.00	20.00		73.33	66.67	8.33	38,70	**40.26***7*
	*0*	*0*		*6.7*	*22.2*	*41.7*	*38.7*	*26.0*
**Summer**	0.000	100.00	73.33					**68.42**
	*0*	*0*	*26.7*					*21.1*

^1^methods in [33] * in numerator - % fish with visible eggs; in denominator - % fish with male gonads (sperm or male).

* in 2 cases: sex not determined

#### Diet of snappers: stomach content analysis

Out of 221 snappers from five species (*L. analis, L. synagris, L. vivanus, O. chrysurus, and R. aurorubens*), from the five studied sites, we found 95 fish with empty stomachs. Many of the fish with empty stomachs were caught in Porto do Sauípe, Bahia. Some fish in Bahia had their stomachs expelled out of their mouths, possibly due to the high depths at which they were caught with the fishermen's hooks. From 126 stomachs that were analyzed, 40% included fish and 42% had crustaceans (Table 4). With the exception of *L. analis*, in which fish was most commonly found in the stomach contents, the other species of snappers ate mostly crabs and shrimp (Table 4). Shrimp is a commonly used bait to catch snappers. Therefore, care should be taken not to overestimate its presence in the stomach contents of snappers caught from hook and line fishing.

**Table 4:** Stomach contents of the five species of snappers (Lutjanidae)

	*L. analis*, Copacabana	*L. synagris*, Bertioga	*L. synagris*, Maceió	*L. vivanus*, P. Sauipe	*O. chrysurus*, P. Sauipe	*R. aurorubens*, P. Sauipe	*L. synagris*, Paraty	Total
**FISH**								
Sardine (Clupeidae)	2				1			**3**
cutlassfish (T*richiurus lepturus*)	1							**1**
another fish (*Haemulon, Scorpaena, Eucinostomus, Diapterus volitans, Muraena sp., Batrachoididae*)^1^	6	7	3	3	12	11	5	**47**

**CRUSTACEAN**^**2**^								
shrimp (*camarão*)		7	3	2	1		4	**17**
crab (*caranguejo*)		2	4					**6**
crab siri (*siri*)		5	3	3	7	1		**19**
tamburutaca		2						**2**
spanish slipper lobster (*lagosta sapateira*)		1						**1**
crayfish (*lagostim*)			1					**1**
another crustacean, rests		1	2				4	**7**

**MOLLUSCS**								
squid (*lula*)	1						1	**2**
mussel (*mexilhão*)	1							**1**
shell of mussel (*concha mexilhão*)	1							**1**
octopus (*polvo*)	1							**1**

**Rest of food**	6	6	4	8	8	6	8	**46**
**Empty**	18	8	2	13	37	1	16	**95**

**Total samples**	**32**	**29**	**15**	**29**	**66**	**16**	**34**	**221**

^1^identification revised by Rodrigo Caires, MZUSP.

^2^the identification of crustacean was revised by Gustavo S. de Melo (MZUSP) as follows: *Callinectes ornatus*, *Callinectes exasperatus*, *Portunus spinimanus, Calappa angusta, Sicyonia *sp., Dendrobranchiata, Isopoda, *Iliacantha subglobosa*, Brachyura sp., Caridea sp., Glyphocrangonidae sp., *Squilla brasiliensis *(Stomatopoda, Squillidae), *Squilla brasiliensis *(Stomatopoda, Squillidae), and *Scyllarus depressus *(Palinuridea, Scyllaridae).

#### Local knowledge: what do fishermen know about snappers?

From a set of interviews that were previously performed in Bertioga, Copacabana, Paraty (SE Brazil), Porto do Sauípe, and Riacho Doce (NE Brazil) (Appendix 1), we selected a sub-sample of fishermen that lived at the study sites and had been fishing at that location for at least ten years. We interviewed a total of seventy fishermen. Their ages ranged between 40 and 60 years old, the number of years they had been fishing ranged between 22 and 48 years and the time they resided at the sites ranged between 27 and 59 years (Table 5).

**Table 5:** Results of interviews performed with artisanal fishers^1^

Fishers and Questions	**Bertioga**^***a ***^**SE Brazil N = 15**	**Copacabana**^***b ***^**SE Brazil N = 13**	Paraty SE Brazil (Praia Grande and Ilha do Araújo) N = 15	Porto Sauípe NE Brazil N = 14	**Riacho Doce**^***c***^**, Maceió NE Brazil N = 13**	Total N = 70
**Fishers interviewed:**						**Range**
***Average Age***	**50**	**49**	**46**	**60**	**40**	**40-60**
Minimum Age	24	25	28	38	27	**24-38**
Maximum Age	80	74	63	73	61	**61-80**
***Average Time fishing***	**32**	**28**	**35**	**48**	**22**	**22-48**
Minimum Time fishing	10	12	24	29	11	**10-29**
Maximum Time fishing	50	55	60	65	50	**50-65**
***Average Local Residence Time***	**27**	**35**	**45**	**59**	**40**	**27-59**
Minimum Local Residence Time	13	10	28	38	27	**10-38**
Maximum Local Residence Time	66	62	63	73	61	**61-73**
**What do snappers eat?**						
***Fish***	5	1	3	11	7	**27**
Sardines (Clupeidae)	2	3	9	5	8	**27**
Manjuba (Engraulidae)	1	6	2	1		**10**
Other fish			Paraty:1	Mackerel: 1 Agullha: 1	Carapau: 4 Mackerel:1 Saramunete: 1	**9**
Caranguejo/siri (Crabs) (caranguejo/siri)	1/			5/1	/1	**6/2**
***Shrimp***	6	8	14	5	2	**35**
Lobster				1		**1**
Other crustacea	1	2				**3**
Marisco (mussels)	3	4		4		**7**
Squid/Octopus		4/	2/	4/2	1/1	**10/3**
Other mollusc	2					**2**
***Algae***	4					**4**
**Where do snappers live?**						
***Rocky substrate***	11	11	12	13	13	**60**
Cascalho (gravel)	1	4	1			**6**
Beaches		1	/3			**1/4**
Coast		1				**1**
Corals		1		1	1	
Deep water	3			2		
**When do snappers are mature/spawn?**						
Does not know	11	7	7	3	8	36
Autumn						0
Winter		2	1		1	4
Spring	3		1	10^*d*^		14
Summer	2	1	5	1	3	12
All year round			1		1	2

^**1 **^Sites: Bertioga, Copacabana, Paraty, Riacho Doce, Maceio and Porto Sauípe, Bahia, Brazil. For this study, we considered fishers with fishing experience and local residence of about 10 years.

^*a*^9 interviews excluded from our sample, ^*b*^5 interviews fishers excluded, ^*c*^2 interviews excluded (fishers with less that 10 years of fishing or local residence)

^*d *^To get a more detailed of answers in Porto do Sauípe, Bahia, 5 fishers explained gonads were mature by August, and that after September-October snappers spawn (11 fishers).

Fish and shrimp is the diet most cited by fishermen for snappers. The fishermen said that snappers live in rocky substrates and spawn in the spring and summer (Table 5). The results from polling the local knowledge, and especially the comparative data for Porto Sauípe, showed that fishermen can very precisely determine the reproductive season of very common species caught in their locality, such as *Lutjanus vivanus *and *Ocyurus chrysusrus *(65-75% of samples with visible eggs in October, 2008, Table 5).

Appendix 1 shown details on fieldwork and interviews (questionnaire and Table 6), on weight and length of the five common snapper species (Tables 7 and 8), on GSI and its statistics (Table 9 and 10), and a reference for the locations (fishing spots) where snappers are often caught by artisanal fishers from each fishing community (Table 11 in Appendix 1).

**Table 6:** Fieldwork for interviews and to collect snappers in 2008 and 2009^1^.

Research site	Trips/no. days for snapper collection	Collection Period	GPS Locations (Google Earth)	Total snappers collected/spp	Total interviews with fishers
Bertioga, SE Brazil	10 (44 days)	April 2008-January 2009	23^0 ^51' 18" 46^0 ^08' 20"	44/4 spp.	24
Copacabana, Rio, SE Brazil	12 (80 days)	April 2008 - March 2009	22^0 ^58' 15" 43^0 ^11' 29"	35/3 spp.	18
Paraty, SE Brazil	02 (7 days)	August and November 2008.	23^0 ^12' 59" 44^0 ^43' 04"	44/3 spp.	15
Porto Sauípe, Entre Rios, Bahia, NE Brazil^2^	02 (6 days)	July and October2008	12^0 ^01' 52" 37^0 ^39' 40"	137/9 spp.	14
Riacho Doce, Maceió, Alagoas, NE Brazil	01 (04 days)	January 2009	9^0 ^33' 50" 35^0 ^39' 21"	28/3 spp.	15

^1^A total of 288 snappers were collected in 142 days, and 86 fishermen were interviewed in the five fisheries. ^2 ^A second trip to Bahia was not initially planned. However, data from fishers on spawning periods collected in our first visit to Bahia made us planning for another trip, in order to double check on the gonad maturation of species. This second trip occurred in October 2008.

**Table 7:** Values of weight distributions the most collected species of snappers

	*L. analis *Copacabana	*L. synagris *Bertioga	*L. synagris*, Maceió	*L. vivanus*, P. Sauipe	*O. chrysurus*, P. Sauipe	*R. aurorubens*, P. Sauipe	*L. synagris*, Paraty
**Mean (g)**	891.9	1174.1	340.0	496.6	488.6	528.8	1245,9
**St. error**	42.6	85.5	38.2	23.9	17.0	64.3	132,3
**Median**	900	1158	300	470	455	440	1625
**St. dev**.	237.4	460.3	147.8	128.5	138.5	257.3	771,2
**Interval**	1000	1620	600	610	780	1000	2350
**Min**	500	480	150	290	320	300	150
**Max**	1500	2100	750	900	1100	1300	2500
**N**	31	29	15	29	66	16	34

**Table 8:** Values of lengths distributions for the most collected species of snappers.

	*L. analis*, Copacabana	*L. synagris*, Bertioga	*L. synagris*, Maceió	*L. vivanus*, P. Sauipe	*O. chrysurus*, P. Sauipe	*R. aurorubens*, P. Sauipe	*L. synagris*, Paraty
**Mean (mm)**	401.0	444.7	282.0	337.9	387.6	351.3	417,8
**St. error**	7.3	10.3	8.6	6.4	4.5	14.8	19,4
**Median**	410	443	270	330	380	335	487,5
**St. dev**.	40.4	55.2	33.4	34.4	36.3	59.3	113.0
**Interval**	220	210	120	160	210	240	305
**Min**	300	350	240	250	320	270	235
**Max**	520	560	360	410	530	510	540
**N**	31	29	15	29	66	16	34

**Table 9:** Mean values of gonadosomatic index (GSI) for species of Lutjanidae (%)

Period	*L. analis *Copacabana	*L. synagris *Bertioga	*L. synagris *Maceió	*L. vivanus *P. Sauipe	*O. chrysurus *P. Sauipe	*R. aurorubens*. P. Sauipe	*L. synagris*, Paraty	Total
**April**	0.55 ± 0.22							**0.55 ± 0.22**
**May**	0.15 ± 0.04	0.85 ± 0.18						**0.36 ± 0.11**
**June**	0.10 ± 0.05	0.52 ± 0.14						**0.45 ± 0.13**
**July**	0.09 ± 0.04			0.45 ± 0.13	0.25 ± 0.03	0.24 ± 0.11		**0.28 ± 0.03**
**August**		0.22 ± 0.12					0.66 ± 0.16	**0.44 ± 0.13**
**September**	0.05^*a*^	1.12 ± 0.84						**0.94 ± 0.71**
**October**	0.07 ± 0.01			1.37 ± 0.25	1.26 ± 0.24	0.45 ± 0.09		**0.94 ± 0.13**
**November**							1.93 ± 0.27	**1.91 ± 0.27**
**December**	0.37 ± 0.27	4.04 ± 0.16						**2.21 ± 1.07**
**January**			3.58 ± 0.46					**3.58 ± 0.46**

**Autumn**	0.34 ± 0.11	0.85 ± 0.18						**0.43 ± 0.11**
**Winter**	0.09 ± 0.03	0.47 ± 0.12		0.45 ± 0.13	0.25 ± 0.03	0.24 ± 0.11	0.66 ± 0.16	**0.32 ± 0.03**
**Spring**	0.07 ± 0.01	1.12 ± 0.84		1.37 ± 0.24	1.26 ± 0.24	0.45 ± 0.09	1.93 ± 0.27	**1.34 ± 0.15**
**Summer**	0.37 ± 0.27	4.04 ± 0.16	3.58 ± 0.46					**3.29 ± 0.43**

^a ^Standard error not possible to calculate - unique value.

**Table 10:** P-values* for pairwise tests of seasonal data on (GSI) of Lutjanidae ^1^

Species, seasons and comparisons				
(a) *L. analis*, Copacabana				
	
	**Winter**	**Spring**	**Summer**	
	
**Autumn**	0.0796	0.0593	0.5942	
**Winter**		1	0.1161	
**Spring**			0.0507	
	
(b) *L. synagris*, Bertioga				
	
	**Winter**	**Spring**	**Summer**	
	
**Autumn**	0.0885	0.1416	0.0641	
**Winter**		0.5528	0.0232	
**Spring**			0.2453	

(c) Comparison, of GSI values between Winter and Spring for four species between Winter and Spring				

	***L. vivanus*, P. Sauipe**	***O. chrysurus*, P. Sauipe**	***R. aurorubens*, P. Sauipe**	***L. synagris*, Paraty**

**P-parameter**	0.0013	0.0003	0.1816	0.3778

^1^using the Kruskall-Wallis statistical test). * if the p-value is less than 0.01(or 0.05) one can assume that the data are drawn from the same distribution.

**Table 11:** Fishing spots used by artisanal fisheries in the five research sites

Research site	Data on fishing sites/spots	Spots and number of fishers citing the spot		
Bertioga, SE Brazil	From Andreoli (2008) interviews (n = 24) and Alcatrazes is confirmed in our snapper sample.	Alcatrazes Island (17 fishers), Montão de Trigo Island (10) Queimada Grande Island (7), and Laje de Santos Island (3), the most mentioned sites.		
Copacabana, Rio, SE Brazil	From our collection.	Cagarras Island (most common in our sample), Laje de Santo Antonio, and Angra dos Reis.		
Paraty, SE Brazil	Marking of fishing spots using GPS Garmin, with the help of fishers Alcides and Marquinhos, November 2008.	Cais da Praia Grande	23°09'06''	44°41'48''
		Ponta da Baleia - Ilha do Araújo	23°09'02''	44°40'55''
		Ponta da Rapada - Ilha Rapada	23°09'33''	44°39'37''
		Ilha dos Ganchos	23°10'25''	44°38'02''
		Laje do Fundo	23°07'54''	44°39'31''
		Laje Rasa	23°07'17''	44°39'13''
		Ilha dos Meros	23°10'57''	44°34'26''
		Laje dos Meros	23°10'40''	44°34'39''
		Laje do Sapê	23°11'00''	44°34'35''
		Laje dos Ganchos	23°10'12''	44°37'12''
Porto Sauípe, Entre Rios, Bahia, NE Brazil	From an interview with the fisher Celinho (66 y. old).	All spots with 140 m deep or more (70 braças): Seladinha, Ponta da Areia, Preto, Selada Grande, Ronco da Caatinga, Sampelício, Caranha, Oco da Galha, Verde, Meio da Vagem, Caça Lobo, Mancha Grande, Amiúda, Caatinga, Verde da Caatinga, Camburú, Verde do Camburú.		

## Discussion

### Size and maturity of snappers

We observed that the five snapper species that were most frequently collected (*Lutjanis analis, L. synagris, L. vivanus, Osciurus chrysurus*, and *Romboplites aurorubens*) were caught at relative early stages of maturity, as exemplified by Figures 3, 4, 5, 6 and 7. *L. analis*, in Copacabana, were caught between 350-450 mm; *L. synagris*, in Bertioga were caught between 350-450 mm and between 470-520 mm in Paraty, and in Riacho Doce, the fish were at sizes ranging between 240-300 mm. *L. vivanus*, in Porto Sauípe, was caught between 300-400 mm; *Ocyurus chrysurus*, in Porto Sauípe, was between 350-400 mm; and *Romboplites aurorubens*, in Porto Sauípe, ranged between 300-350 mm. The recorded length for maturity of those snapper species are recorded in Froese and Pauly (2010). The lengths for maturity are as follows: *L. analis*, 510 mm; *L. synagris*, 236 mm; *L. vivanus *518 mm; *O. chrysurus*, 245 mm; and *R. aurorubens*, 200 mm. Growth values (L_max_) for *L. analis *(850 mm), *L. synagris *(650 mm), and *L. vivanus *(750 mm) were found in NE Brazil [13]. This information reinforced the observation that while some of the studied local artisanal fisheries have been catching fish within a reasonable size, such as *L. synagris *in Bertioga and Paraty, different patterns occur at other sites. Those differences occurred for *L. analis *in Copacabana, for *L. synagris *in Riacho Doce and for *L. vivanus *in Porto Sauípe, which were caught before reaching maturity. In particular, the situation for *L. analis *is problematic because it is considered a vulnerable species[61].

Additionally, particular attention is needed for the species *L. synagris*, since there are other studies showing catches of snappers in juvenile stages in NE Brazil [14]. According to the cited study, artisanal fisheries using boats such as rafts ('jangadas'), and other small boats locally named 'paquetes', used in shallow waters, could be probably impacting populations of L. s*ynagris*. Our results reinforce this information, since our findings reveal that *L. synagris *was being caught too early, still in its juvenile stages (Figure 3) before maturity (236 mm) [10] in the shallow waters of Riacho Doce, Maceió. Such results might indicate overfishing (decreasing size of catches), but we still cannot determine whether the cause of that impact is derived from the local artisanal fishing or was a result of industrial fishing.

Knowledge on reproductive periods of species of snappers is an useful information towards fishery management. Results from interviews indicate that some fishermen know about the reproduction species (a half does not know about the reproductive behavior of species). Considering the site where there is the highest occurrence and diversity of species of snappers on artisanal landings (Porto Sauípe Bahia), we noticed that knowledge on reproduction of snappers is higher among the old fishermen (averaging an age of 63, n = 11); fishermen that do not know about snapper reproduction aged an average of 48 years old (n = 3). Therefore, suggestive periods for fishing snappers, and for closed season, avoiding thus reproductive periods, could be obtained by interviewing especially older fishers, that could help directly in management.

It is important to address that, in spite of the significance of artisanal fishing in Brazil [23], and the importance of snappers and other reef fishes as commercial catches [13,14] there is no legislation that regulates the size or number of the snappers that are caught. Additionally, the economic importance of snappers has led them to a status of exploited populations [14]. After consulting the federal legislation of IBAMA (http://www.pescamadora.com.br/peixes_agua_salgada/tamanho_minimos_peixes_agua_salgada.pdf) we did not find any minimum threshold for catching snappers (Lutjanidae) in Brazil. What we do not know, however, is why the fish in Copacabana (Rio) and in NE Brazil (Riacho doce) were being caught so early. It may be due to the impact of artisanal fisheries, or it is possible that artisanal fisheries are only able to catch the fish that have been not captured by industrial fisheries.

### Local knowledge, management, and target fish

The similarity between the information from the relatively scarce biological literature on snappers (Lutjanidae) and the information provided by fishermen was striking. Fish and crustaceans were the main food items of snappers according to the literature as well as the fishermen [10,62-67].

According to the interviewed fishermen, many snappers spawn in the spring and live in rocky substrates (Table 5). The results of our biological survey indicated that snappers have a higher GSI, which indicates reproductive activity, and show more individuals with visible eggs in the spring in Porto Sauípe (Table 3). These results reinforce the need to include local fishermen in biological research. As knowledge on fish reproduction in the scientific literature is generally scarce, the clues fishermen give can be useful for defining periods of closed fishing activities. Most results on snappers identifies the spring and the summer as spawning periods. A suggestion given by this study would be to identify, together with fishermen, the spawning periods more claearly per species of snapper. It worth paraphrasing Thresher (1984: 121)[68]:

Spawning for most tropical snappers seems to occur over a large part of the year and may take place year-round for many species. Spawning peaks, however, generally coincide with periods of warm water temperature, though not necessarily the warmest part of the year. In the Western Atlantic, for example, spawning reaches a peak in the summer near the northern limits of the family's range (refs, not cited here), but peaks in spring or its bimodal with peaks in the spring and fall in the tropics.

Finally, besides the importance of the spring and summer as reproductive seasons for snappers at the studied sites along the Brazilian coast, some snappers form spawning aggregations, such as the Lane snapper, *L. analis *[69] important to consider for the management of the fisheries. This species is considered overexploited in Brazil, along with the other snappers *L. synagris *and *O. chrysurus *[54].

Fishermen from Porto do Sauípe showed the most knowledge of snappers' reproduction (Table 5), compared to fishermen from the other sites. Actually, these fishermen encouraged us to return to Porto do Sauípe to collect mature snappers during the correct season. We had one trip planned to Porto do Sauípe (July 2008), but information gathered from the interviews lead us to return to Porto do Sauípe in October 2008 because many fishermen stated that we would find mature fish at that time (Table 5). As the fishermen said, most of the fish with visible eggs were observed during October in Porto Sauipe (Appendix 1). From all of the sites, Porto do Sauípe was the one with the most available snappers [53], and snapper is a very common catch at this site. For fishermen in that area, snappers are their target species. This result shows that fishermen's knowledge is usually directed at target fish species.

Target species are the ones that are most manipulated (caught, cleaned, consumed and sold) by fishermen, and thus fishermen are more knowledgeable about these species. These results are important for considering ethnobiological studies in general, especially when trying to use local knowledge for fishery management. Improving the dialogue between fishermen and managers could be done by a co-management, engaging researchers in a careful discrimination of ethnobiological results, as already suggested [31]. It would be better to rely on the knowledge that fishermen have on the target fish, and not all fish in general, as an ethnobiological approach towards local management. Etnobiological approaches are necessary in data less fisheries, as are mostly artisanal fisheries in Brazil, and focusing on target species associated with fishermen turns data collected for management more reliable, since fishermen know more on target species.

Another important observation from our study was that fishermen possessed accurate, detailed knowledge of the diet of snappers because of the bait they use to catch these fish. The fishermen's knowledge correlated with data from the literature in that snappers feed basically on crustaceans and fish [9,11,64,65,70]. When observations given by fishermen (Table 5) are compared to our samples of stomach contents (Table 4), we found that we could rely on the fishermen's information regarding the diet of snappers. Diet is important for management purposes because, if some areas are to be preserved for fishing in the future, it is wise to determine the areas where the fish and crustaceans that snappers consume are found. Other studies have shown that coastal and freshwater fishermen have detailed knowledge of fish diets that largely agreed with the biological literature. The fishermen's knowledge could a useful resource for understanding the ecological interactions among exploited species and the effects of fishing on food chains [34,37,71,72].

### Fishermen's motivations for managing artisanal fisheries

In terms of fishery management for reef fishes such as snappers, it is important to analyze the factors that motivate fishermen to catch fish species at early stages of maturity. Fishermen are often poor, rural people in Brazil, and they are dependent on fishing to sustain their families. This imposes the classic dilemma of how conservation could be made attractive to poor fishermen [23]. This dilemma is exacerbated by the reduction of fishing areas for artisanal fishermen, since they are squeezed between protected areas and sites used by industrial fisheries.

Artisanal fishermen from the coast of Brazil have been pressured in terms of their use of the marine space by environmental government agencies through the establishment of top-down conservation areas (without consultation or participation of locals or users) [73]. In addition, there is also conflict between industrial and artisanal fisheries competing for space [55]. These conflicts may even push artisanal fishermen to less conservative behaviors, since they can feel stimulated to obtain higher catches, or even to enter protected areas, before trawlers from industrial fisheries come into that areas[74].

Current literature has stressed the economic mechanisms behind the activities of fisheries, and in particular, the subsistence and sustainability of artisanal fisheries. For example, economic drivers are an important part of fishery management [75] (page 12163):

"For successful fisheries management, it will be necessary to move beyond the symptoms of fishing and to take into account drivers of harvest pressure that result in potentially significant ecosystem change. One step in this direction is to incorporate leading indicators for current and future impacts of fishing into management. What motivates fishermen?"

The access to resources, the importance of local rules, the equity in terms of access, along with the necessity for fishermen to sustain their families [23], are variables that must be considered in management propositions. Otherwise, the inshore reef fisheries will continue to be vulnerable marine fisheries. Two participatory categories can be drivers for fishermen to participate in management processes: the valorization of their local knowledge on fish species, as well as compensatory mechanisms, as can be 'payments for environmental services' (PES).

The first, the use of local knowledge, is a very stimulating process to fishermen, since they feel motivated to talk about fish and about the aquatic space (fishing spots, sites, islands). Finding mechanisms of obtaining data from fishermen, embedding them into processes of management, training them for monitoring processes, thus putting '*both knowledges' *(scientific and local) as an interactive process, can motivate fishermen to be interested in conservation, and into co-managing the fishery.

The suggestion for compensatory mechanisms, such as payment for environmental services, could help driving the interest of fishermen towards conservation, thereby avoiding or reducing the current fishing of immature snappers. Payments for environmental services (PES) are voluntary transactions that involve well defined environmental services. These environmental services are purchased by a service buyer from a service provider if and only if the service provider secures the service provision (that is a condition) [76]. In our case, local fishermen could be paid to monitor fishing sites used by industrial fisheries and to provide information about the landing of snappers at their local fisheries, since fish landing data for snapper species is incomplete [22]. Fishermen could contribute to this data if they are included in management processes.

PES could be a practical road in order to have protected areas for biodiversity conservation and co-management processes. Mechanisms of payments for fishermen to avoid fishing in protected areas and to help monitoring those sites were suggested for other artisanal fishery in SE Brazil (Arraial do Cabo, Rio de Janeiro), by using an already existent payment mechanism in Brazil, the 'defeso' [77]. The 'defeso' is a governmental payment for fishermen in periods of closed shrimp fishing. Such type of payment we suggest could be applicable for payments for fishermen avoiding closed protected areas, for example, or closed periods when some snapper species are spawning.

## Conclusions: management and research suggestions

Artisanal fisheries are important in the commercial fisheries of tropical countries, especially in Brazil. Snappers are target species, having good commercial value, but are in relative danger of being overfished. Some of the species that are caught, such as *Lutjanus synagris *in Riacho Doce, Maceió, and *Lutjanus analis *in Copacabana, Rio de Janeiro, have been caught at sizes below the minimum maturity length.

A dilemma exists when facing conservation and tropical artisanal fisheries, since many fishermen are poor and depend upon fishing for their livelihoods. The other dilemma that runs against conservation, looking through fishermen lens, are trawlers that enter bays and coastal shallow areas, sweeping out the fishing spots, pushing artisanal fishermen to see conservation as a responsibility thrown out on their shoulders. Facing these dilemmas, managers should make use of mechanisms that integrate local fishermen's knowledge into fisheries management as proposed by previous surveys. In this study, we propose: 1) that local fishermen have viable knowledge of the diet of snappers and of their reproductive season, which could be used for management purposes in a dialogue process with managers and academics; 2) that such a dialogue should rely on target species because fishermen tend to have more knowledge on the commonly caught species; 3) that motivation should enhance strategies for conservation in countries were artisanal fishing is very relevant, and where impoverished people depend upon those resources. To deal with poverty, we propose the ecological-economic strategy of paying for environmental services. Such proposition was already given concerning the management of the Arraial do cabo fishery, in Rio de Janeiro [77]. Our suggestions are specified as follows:

1) Co-management of fishing sites could enhance the participation of artisanal fisheries in management processes and conservation. One hypothesis is that, if the major impact on snapper fisheries comes from industrial fishing and artisanal fisheries are getting the leftover fish, which are juveniles, then this approach could minimize overfishing. The co-management of reef areas (between fishermen and the environmental agencies, for example) seems an alternative since fishermen know about the fishing spots of snappers and have some relative knowledge on the reproductive period of snappers.

*2) *Management should be especially focused on mutton snapper (*L. analis*, vermelho-cióba or cióba) and lane snapper (*L. synagris*) because they have been appeared on landings at juvenile stages. We can suggest closed fishing seasons for these species, such as in the spring and part of the summer at Copacabana. Other closed seasons can be negotiated with fishermen from Porto do Sauípe, per species, since this is the the most productive site for catching snappers, compared to the other sites we studied.

*3) *Finally, processes for payments for environmental services are suggestions that could help fishers to manage resources. In Copacabana, Rio de Janeiro, there is an urgent need to manage the *L. analis *population; in Riacho Doce, Maceió, and in Porto do Sauípe, the diversity and importance of snapper in artisanal fisheries justify such an initiative. Payments for environmental services could be directed through fishing accords or agreements (a negotiation process that already occur in Brazil) (23). By incorporating payments for environmental services, fishermen could be motivated to help transform the top-down approach in Brazilian fisheries into a more participatory process that works toward the conservation of marine resources.

## Competing interests

The authors declare that they have no competing interests.

## Authors' contributions

AB conceived the study, collected data in the fieldsites, organized, and analyzed data on snappers, besides writing the paper; SS organized and analyzed quantitatively part of the data collected; LGA collected data in Paraty site; LECO collected data in Paraty and Riacho Doce sites; MC collected data in Copacabana, CMM collected data in Riacho Doce, AGF and TBA collected data on Bertioga, and RAMS contributed in the methods and in analyzing results. All authors read and approved the final manuscript.

## Appendix 1

### Questionnaire Protocol

Questions on snappers:

1) Which snappers occur here?

2) What they eat?

3) Where (substract) they live?

4) When they are mature? (gonads)

Questions about the fisherman:

1) Fisherman name

2) Age

3) Study Site

4) Number of years fishing

5) Number of years of residence
